# Recovery of Carbon Fibre from Waste Prepreg via Microwave Pyrolysis

**DOI:** 10.3390/polym13081231

**Published:** 2021-04-10

**Authors:** Siqi Hao, Lizhe He, Jiaqi Liu, Yuhao Liu, Chris Rudd, Xiaoling Liu

**Affiliations:** 1 Faculty of Science and Engineering, University of Nottingham Ningbo China, Ningbo 315100, China; Siqi.HAO@nottingham.edu.cn (S.H.); Lizhe.He@nottingham.edu.cn (L.H.); zy21709@nottingham.edu.cn (J.L.); suxyl1@nottingham.edu.cn (Y.L.); 2 New Material Institute, University of Nottingham Ningbo China, Ningbo 315100, China; 3 James Cook University (JCU), Singapore 387380, Singapore; chris.rudd@jcu.edu.au

**Keywords:** carbon fibre, recycling, microwave, pyrolysis

## Abstract

Management of waste from carbon fibre composites has become a significant societal issue as the application of composite grows across many industries. In this study, carbon fibres (CF) were successfully recovered from cured carbon fibre/epoxy (CF/EP) prepreg under microwave pyrolysis at 450, 550 and 650 °C followed by oxidation of any residual char. The recovered fibres were investigated for their tensile properties, surface morphologies and the elements/functional groups presented on the surface. The chemical compositions of gaseous and oil pyrolysis products were also analysed. The microwave pyrolysis effectively pyrolyzed the epoxy (EP) resin. Char residue remained on the fibre surface and the amount of char reduced as the pyrolysis temperature increased. Compared to virgin fibres, the recovered fibre suffered from a strength reduction by less than 20%, and this reduction could be mitigated by reducing the pyrolysis temperature. The surface of recovered fibre remained clean and smooth, while the profile of elements and functional groups at the surface were similar to those of virgin fibres. The main gaseous products were CO, H_2_, CO_2_ and CH_4,_ whilst the liquid product stream included phenolic and aromatic compounds.

## 1. Introduction

Carbon fibre reinforced plastic (CFRP) composite has been widely used in automotive industries, wind energy industries and other engineering applications due to its unique combination of low-weight, high elasticity and strength and good corrosion resistance [1,2,3]. The excellent mechanical properties of carbon fibre reinforced thermoset composites and their increasing affordability means that industrial demand continues to grow rapidly [4]. However, the continuing increase in widespread applications of CFRP has led to much waste consisting of End-Of-Life (EOL) components and manufacturing waste (prepreg offcuts, obsolete moulds, faulty parts, etc.), making recycling as one of the vital issues in composite industries [5].

Thermosetting resins impose a particular challenge to CFRP recycling since they cannot be re-melted and re-shaped. To deal with the increasing thermoset composite waste, recycling technologies including fluidised bed processes, solvolysis and pyrolysis have been proposed and demonstrated technical feasibility [6]. In a fluidised bed process, the waste composite is heated using air at a temperature around 450–550 °C in a bubbling sand bed. The fibres are separated from organic exhausts using a cyclone [6,7,8]. The major advantage of this process is being capable of dealing with contaminated composite wastes, but the polymer matrix is only burnt off for energy recovery. During solvolysis, chemical dissolution reagents are used to depolymerise the resin matrix [9]. Solvolysis enables the recovery of clean fibres as well as recycles the thermoset matrix as a monomer phase [10]. However, solvolysis remains energy intensive and requires organic solvents such as ethanol or propanol. The process is limited to laboratory scale, and it is difficult to generalise solvent selection for mixed waste streams [11].

Pyrolysis is one of the most widespread recycling processes for CFRP waste and is available on an industrial scale. Here, both fibre and thermoset resin are recovered by heating waste composite in an inert gas environment at a temperature between 450 and 700 °C [12]. The resin is converted into pyrolytic gas, oil and solid char. The pyrolytic gas and liquid are collected via a condensation stage that allows them to be reused as potentially valuable chemicals [13]. The pyrolytic char, however, remains on the surface of the recovered fibre, inhibiting their reuse options. A subsequent oxidation is typically essential to remove the char residues and obtain clean fibres. Recent advances in pyrolysis technology have led to the establishment of several industrial-scale recycling operations, mainly in Europe and North America. However, conventional pyrolysis always requires a large amount of energy or long processing times. Regarding this problem, radiofrequency or microwave heating has emerged in several studies. With the advantages of rapid and selective heating [14], microwave heating shows the potential to yield clean carbon fibres from a thermoset composite waste stream. Lester et al. [15] confirmed the feasibility of recycling carbon fibre with microwave heating using a 3 kW magnetron device. The recycled fibre retained 80% of the tensile strength of virgin fibres. Jiang et al. [16] recovered carbon fibres via microwave heating and re-used them as short fibre reinforcement in thermoplastic composites. The recycled carbon fibre provided superior mechanical properties in (non-polar) polypropylene-based composites, while the virgin fibre performed better in the (polar) polyamide matrix. Obunai et al. [17] studied the effect of different gas atmospheres on the mechanism of carbon fibre recycling under microwave heating, as well as the mechanical properties of recycled carbon fibre. Argon was the most conducive atmosphere for recovering clean fibre without damage. The authors also proposed the mechanism of microwave separation, whereby the fibre was first heated via the antenna effect by microwave irradiation to gasify the resin, and then gasified resin was further decomposed by spark glow plasma between carbon fibres. Deng et al. [18] compared carbon fibres recycling via microwave thermolysis and traditional thermolysis under a pure oxygen atmosphere and found 450 °C to be the optimum temperature. They also reported that microwave thermolysis saved 57% reaction time and increased recovery ratio by 15% compared to conventional thermolysis. Whilst there is an understandable focus on the yield and condition of the (high value) fibre product in composite recycling, little work is reported on the other pyrolytic products from the thermoset resin.

In this study, the cured CF/EP prepregs were pyrolyzed using the microwave pyrolysis technique under different temperatures. The product yield, tensile properties, surface morphologies and surface functional groups of the recovered CF were investigated. In addition, the composition of the pyrolytic gas and oil was also characterised. 

## 2. Materials and Method

### 2.1. Materials

Unidirectional carbon fibre reinforced epoxy (CF/EP) prepregs consisting of Toray@ T700 carbon fibre (63 wt.%) and Bisphenol-A epoxy resins (37 wt.%) was supplied by Aojing^@^ Composite Company, Shanghai, China. The CF/EP prepreg was cut to a size of 200 mm × 200 mm and then cured in an atmospheric oven at 120 °C for 2 h. The prepreg was collected and stored in a dry environment before use. Dichloromethane (CH_2_Cl_2_, purity: 99.99%, Sino Chemical Reagent, Shanghai, China) was used as a solvent in the condensation section to collect the pyrolytic oil. A higher grade of CH_2_Cl_2_ (Chromasolv^@^ Plus for HPLC, Sigma-Aldrich, Shanghai, China) was also employed as a solvent for gas chromatograph–mass spectrometry (GC-MS) analysis.

### 2.2. Microwave Pyrolysis Recycling

**Figure 1:**
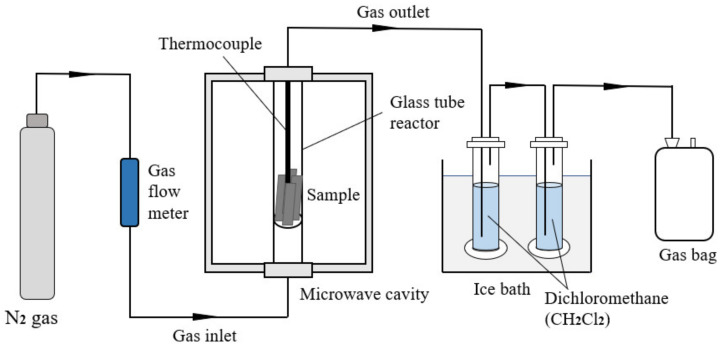
The experimental setup for the microwave pyrolysis system.

Microwave pyrolysis of the cured prepreg was carried out in a 2.45 GHz multi-mode microwave cavity (Jiequan Microwave Co., Ltd., Nanjing, China) with the maximum power output of 3 kW, using the experiment set-up summarised in Figure 1. The pyrolysis was carried out under the temperature control mode. Materials were first heated from room temperature to a determined temperature at a constant heating rate, followed by an isothermal dwelling period, during which the power output of the microwave radiation was regulated via a PID controller. For each test, 20 g of prepreg was trimmed into 150 mm × 50 mm specimens and inserted into the inner glass tube reactor. An insulated K-Type thermocouple (time constant < 24 s) was inserted into the centre of the tube to monitor the temperature. A nitrogen gas flow at the rate of 100 mL/min was then used to purge the chamber for 10 min and provide an inert nitrogen atmosphere for pyrolysis. The specimens were heated via two 1.5 kW magnetrons to the chamber temperature of 450, 550 and 650 °C and isothermally held for 15 min. After microwave pyrolysis, the samples were collected and heated in air at 550 °C for 30 min. The experimental schema is presented in Table 1. After oxidation, the fibres were collected for further characterisation. 

### 2.3. Characterisation

#### 2.3.1. Thermogravimetric Analysis (TGA)

A ramping test was carried out to determine the thermal degradation behaviour of the prepreg under nitrogen and air. The samples were heated from room temperature to 1100 °C at 10 °C/min using a thermogravimetric analyser (SDT Q600, TA Instruments^®^, New Castle, DE, USA). The volatilisation temperature of the prepreg in nitrogen and the oxidation temperature in the air were determined from the TGA curves and their first derivative (DTG) corresponding to ramping test carried out in nitrogen and air.

#### 2.3.2. Pyrolytic Product Yield Analysis

The yield of solid pyrolytic product was determined from the mass of solid residue inside the glass tube reactor. The liquid product consisted of oils absorbed by the two gas-washing bottles and that remaining on the inside of the glass tube reactor. The oils were collected by evaporating the dichloromethane in a fume hood. The yield of liquid product was then determined from the total mass of oil collected. The assumed gas product yield was estimated from the difference in resin weight percentage with solid and oil product yield.

#### 2.3.3. Single Fibre Tensile Strength (SFTT)

The tensile strength and modulus of virgin and recovered fibres were characterised using the single fibre tensile tester (DiaStron, Hampshire, UK) and conducted following ISO 11566 *Carbon fibre properties determination standard* [19]. The carbon fibres were bonded onto plastic tabs with UV-curable resin (Dymax Ultra Light-Weld^®^ 3193, Wiesbaden, Germany). After measuring the fibre diameter via laser diffraction (LDS0200 module), the tensile properties of the carbon fibres were measured using a high-precision extensometer (LEX820 module) with a 20 N load cell. A constant strain of 1 mm/min was applied to the fibres with 4-mm gauge length, and the stress–strain curves of the mounted fibres were recorded automatically. Thirty replicates were tested for each type of carbon fibre.

#### 2.3.4. Scanning Electron Microscopy (SEM)

The surface morphologies of the virgin and recycled carbon fibre were observed using a scanning electron microscope (Sigma VP, Zeiss^®^, Oberkochen, Germany) to investigate the degree of degradation of the resin matrix and potential damage to the fibre surfaces [20]. The operating accelerating voltage was 10 kV and the working distance was 9.5–10 mm. Secondary electron detector (SE2) and In-lens detectors were applied for SEM imaging. 

#### 2.3.5. X-ray Photoelectron Spectroscopy (XPS)

The surface element and functional group of virgin and recycled carbon fibre were determined using X-ray photoelectron spectrometer (XPS, Axis Ultra Dld, Shimadzu^®^, Kyoto, Japan) with an Al Kα X-ray source (1486.6 eV) at a power of 15 kV and 450 W. The wide scan spectra with a step of 1 eV and high-resolution spectra with the step of 0.05 eV were recorded in the range of 0–1200 eV. Surface atomic composition and curve-fitting of the XPS spectrum were analysed using CasaXPS software. The carbon (C), hydrogen (H), oxygen (O) and nitrogen (N) traces were scanned. The binding energy was calibrated by referring to the C 1s peak at 284.8 eV [21].

#### 2.3.6. Gas Chromatography (GC)

The composition of the pyrolytic gas produced after microwave heating was determined using a gas chromatograph (GC, GC-2014, Shimadzu^®^, Kyoto, Japan) equipped with 1 FID detector, 2 TCD detectors, 8 molecular sieve columns and 1 HP-AL/s capillary column. The injection temperature, column temperature and detectors temperature were set at 60, 200 and 170 °C, respectively. The system GC is capable of separating C1–C5 hydrocarbons (CH_4_, C_2_H_6_, C_2_H_4_, C_3_H_8_, C_3_H_6_, C_4_H_10_, C_4_H_8_, C_4_H_6_, i-C_5_H_12_ n-C_5_H_12_, etc.) and permanent gas (O_2_, N_2_, H_2_, CO, CH_4_, CO_2_, etc.) simultaneously; their volume contents can also be determined using the external standard method. 

#### 2.3.7. Gas Chromatography-Mass Spectrometry (GCMS)

The composition of pyrolytic oil was analysed using gas chromatograph-mass spectrometry (GC-MS, Agilent 7890–5975C, Santa Clara, CA, USA). An HP-5 ms capillary column (30 m length, 0.25 mm internal diameter, 0.25 μm film thickness) was used. The initial oven temperature of the GC was kept at 60 °C for 2 min before increasing to 280 °C at a heating rate of 10 °C/min, followed by 2 min of dwell. The chromatograph was equipped with a split/splitless injector used in the split mode, with a split ratio of 50:1. Peaks of the chromatogram were identified by comparing their mass spectra with the NIST 2011 library database. 

## 3. Results and Discussion

### 3.1. Thermogravimetric Analysis of Carbon Fibre Prepreg

**Figure 2:**
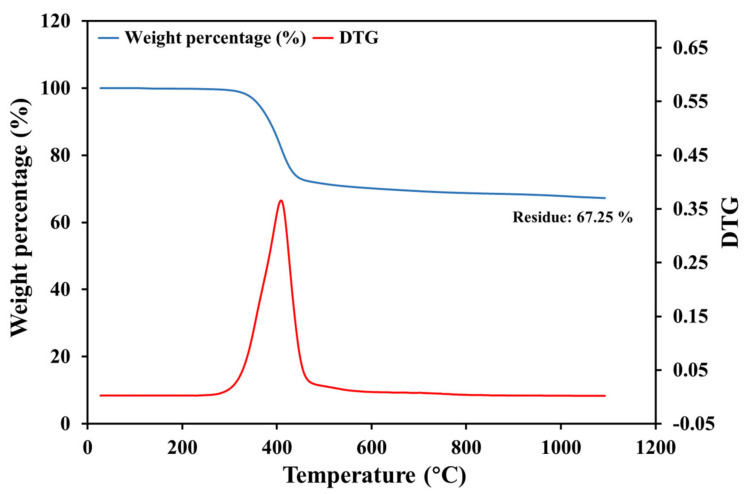
Thermogravimetric curves of CF/EP prepreg heated from room temperature to 1100 °C in nitrogen.

Figure 2 shows the TGA and DTG curve of prepreg samples heated in nitrogen. The mass of specimens reduced significantly at 300–400 °C, corresponding to the thermal degradation of the epoxy matrix. The overall mass loss at 1100 °C was 32.75 wt.%, which was lower than the nominal resin content of the prepreg (37 wt.%). This difference was attributed to the pyrolytic char generated during the degradation of the aromatic and cyclic structure in the epoxy resin [22]. This also implied that the pyrolysis in nitrogen was unlikely to yield clean fibres without char [3]. 

**Figure 3:**
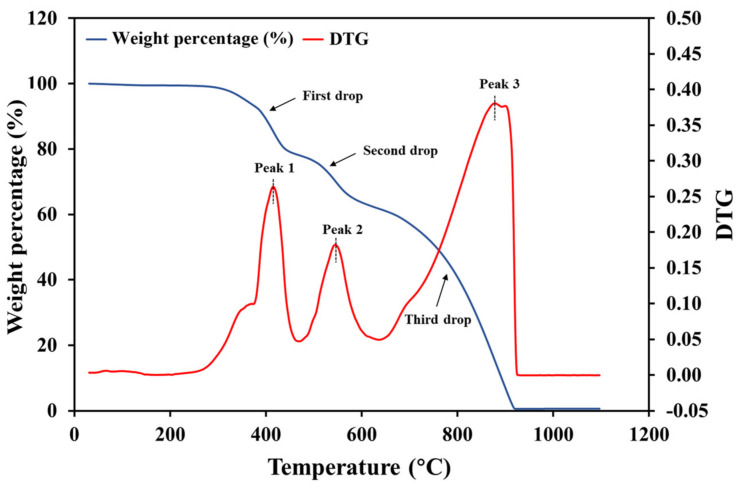
Thermogravimetric curves of CF/EP prepreg heated from room temperature to 1100 °C in air.

Figure 3 shows the TGA and DTG curve of prepreg samples heated in air. Both curves exhibited three main stages of the pyrolysis, with the corresponding mass losses of 21 wt.%, 16 wt.% and 61 wt.%, respectively. The final residue mass was close to zero, indicating that the composite was completely pyrolyzed in the air. The three corresponding peaks occurred in the temperature ranges of: (a) 250–450 °C; (b) 450–650 °C; and (c) 650–900 °C. The sharp peaks at around 415 °C in the DTG curves obtained from heating in either nitrogen or air. This peak was attributed to the pyrolysis of epoxy. The pyrolytic char was oxidised between 450 and 650 °C, with the most intensive char oxidation occurred at around 550 °C. Upon heating above 650 °C, the carbon fibre was oxidised, and the residual mass dropped to 0 at 950 °C. In summary, when heated from room temperature to 1100 °C in the air atmosphere, the pre-preg samples underwent: (a) oxidation of the epoxy resin; (b) oxidation of the char; and (c) oxidation of carbon fibre [23]. The TGA results indicate that the temperature should be carefully selected to eliminate pyrolytic char whilst minimising fibre oxidation. 

### 3.2. Yield of The Pyrolytic Product after Microwave Pyrolysis

To investigate the effect of temperature on the extent of resin pyrolysis and quality of recycled carbon, the temperature of subsequent microwave pyrolysis study was set at 450, 550 and 650 °C. Pyrolysis was carried out in nitrogen to avoid the oxidation of recovered carbon fibres.

The pyrolytic solid residue, liquid and gas yields obtained at different microwave pyrolysis temperature are listed in Table 2. The temperature had a positive effect in reducing the char content as well as increasing the yield of oils and gaseous products. As the temperature increased from 450 to 650 °C, the char residue reduced from 6.1 wt.% to 2.9 wt.%, while the yield of liquid and gas products increased. These findings were also reported in previous works investigating the pyrolysis of pure epoxy [24] and thermolysis of carbon fibre reinforced polybenzoxazine resin composite [25], while the lower char residue correlated to elevated pyrolysis temperature was similarly reported in studies of the pyrolysis process of particle coal [26], biomass [27] and waste [28]. A possible explanation is that the higher temperature provided greater thermal energy to break down the strong organic bonds in the epoxy matrix [29]. Meanwhile, an increasing gas yield is reportedly associated with increased cracking of volatiles at higher temperatures, which is attributed to the thermal cracking of C–C bonds produced at higher temperatures [30] and conversion of epoxy into volatiles, such as hydrogen, methane, carbon monoxide and carbon dioxide [31]. Regarding the pyrolysis of CF/EP composites, a higher microwave pyrolysis temperature led to the lower char residues at the surface of the recovered carbon fibres.

### 3.3. Tensile Properties of Recycled Carbon Fibres

The recovered and virgin carbon fibres were tested for their tensile properties to understand the change of mechanical performance after the microwave pyrolysis. As shown in Table 3, the Weibull tensile strength and tensile modulus of virgin carbon fibre are 4670 MPa and 212.22 GPa, respectively. With increasing pyrolysis temperature, the recovered carbon fibres exhibited 13–20% lower tensile strength. The tensile modulus of the recovered carbon fibres was also slightly (<10%) lower than that of virgin fibre.

The degradation in tensile strength was correlated to the pyrolysis process as well as the subsequent char oxidation process [32]. The reduced tensile strength with increased pyrolysis temperature, as similarly reported by López et al. [25] and Ma et al. [33], was possibly correlated to the char residue presented at the surface of recovered fibres. As shown in Table 2, the char content reduced with increasing temperature. A higher char content would better protect the fibres from being oxidised during the char oxidation process, thus preserving superior fibre tensile strength. 

The tensile properties of carbon fibres recovered from various technologies have been well documented, as mentioned in the above references to the work of Lester [15] and Obunai [17]. Other studies report catastrophic strength reduction of recovered carbon fibres. Emmerich’s study of CFRP using microwave heating for 180 min at 600 W showed only 24% strength retention [34]. Similarly, Pimenta and Pinho reported 21% and 84% reductions in fibre diameter and tensile properties respectively, for carbon fibres recovered from the conventional pyrolysis process [35]. The present high level of strength retention (~90%) demonstrates the technical feasibility of recycling high quality carbon fibre via microwave pyrolysis [36].

### 3.4. Surface Morphology of Recovered Fibres

**Figure 4:**
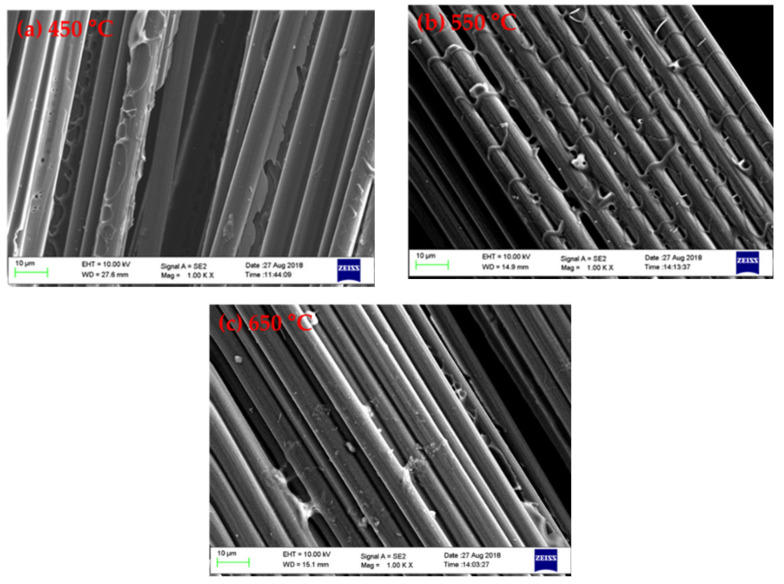
The surface morphology of carbon fibre after microwave pyrolysis at: (**a**) 450 °C; (**b**) 550 °C; and (**c**) 650 °C.

The surface morphologies of the carbon fibre after pyrolysis are shown in Figure 4. As the pyrolysis temperature increased, less char was retained on the fibre surface, and consequently the carbon fibres were less bound by char residues. These findings are consistent with the calculations of char yield, as well as our previous work reporting the reduced content of pyrolytic char with higher pyrolysis temperatures [37]. 

**Figure 5:**
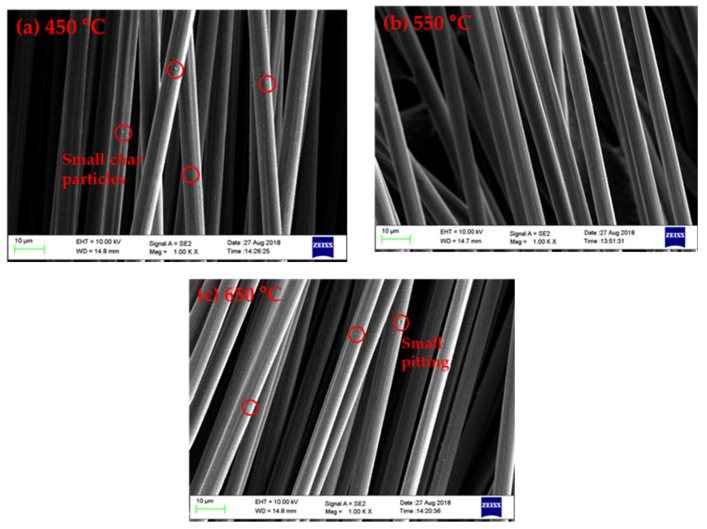
The surface morphology of recycled carbon fibre after oxidation. Fibres were first microwave-pyrolyzed at: (**a**) 450 °C; (**b**) 550 °C; and (**c**) 650 °C.

Figure 5 shows the surface morphologies of recovered carbon fibre after oxidation. In general, the recovered fibres exhibited a relatively clean and smooth surface without being damaged, which further agrees with previous work [38]. The surface morphology of carbon fibres recovered from pyrolysis at 550 °C was similar to those of virgin fibres. In contrast, a small amount of char and ash were observed on the fibres pyrolyzed at 450 °C [39]. Some pitting was evident on the fibre pyrolyzed at 650 °C, possibly due to excessive oxidation. These finding also support the previous hypothesis that the char residue protects the carbon fibres from the worst of oxidative damage.

### 3.5. Surface Element and Functional Groups of Recycled Fibres

**Figure 6:**
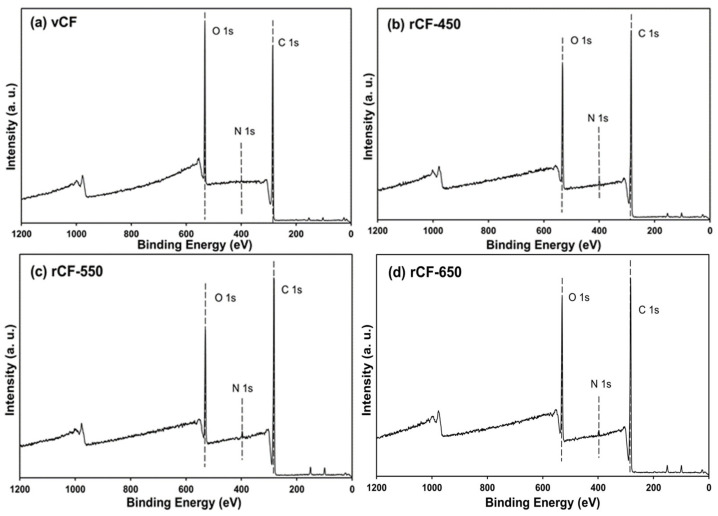
XPS wide-scan spectra of: (**a**) vCF; (**b**) rCF-450; (**c**) rCF-550; and (**d**) rCF-650.

The profiles of chemical elements and functional groups on the surface of carbon fibres were investigated using XPS. The element and composition on carbon fibre surfaces were analysed from the wide scan spectra (Figure 6) and listed in Table 4. As shown in Figure 6, two wide-scan spectra contain two main peaks, which correspond to carbon (280–292 eV) and oxygen (525–540 eV) [39]. Trace amounts of nitrogen and silicon were also detected, which potentially arise from additives (e.g., sizing agents) applied in the carbon fibre manufacturing process [40]. Table 4 shows the concentration of the main element on the carbon fibre surface. The atomic contents of carbon, oxygen and nitrogen of the virgin CF were 77.10%, 22.39% and 0.51%, respectively, with the O/C ratio at 0.2904, which corresponds to the O/C ratio of commercial epoxy resin (0.29) rather than that of virgin carbon fibres (0.2) [7]. This result implies that the virgin carbon fibre surface was sized with epoxy.

The recovered carbon fibre showed similar elemental compositions to the virgin carbon fibres but differed in their atomic ratios, probably due to the incomplete carbonisation of the precursor or surface treatments at the end of the fibre manufacturing process [41]. As such, the removal of sizing on recycled carbon fibre was inferred from the increased nitrogen proportion. The O/C ratio is a quantitative measurement of oxygen-containing functional groups on carbon fibres surface and a good indicator of the effective surface area of chemical bonding between carbon fibres and resins [7]. The presence of C reduced while that of the O increased gradually for recycled fibre as the pyrolysis temperature increased from 450 to 650 °C, leading to an increased O/C ratio. This finding was also reported by other researchers [25,39,42]. A higher O/C ratio implied that some oxygen-containing functional groups were introduced onto the surface of recovered carbon fibre, which may potentially enhance the adhesion between recovered CF and polymer matrix [43]. As the pyrolysis temperature increased from 450 to 650 °C, the O/C ratio increased from 0.253 to 0.331, indicating that a higher pyrolysis temperature promotes the formation of oxygen-containing group on the fibre surface. 

**Figure 7:**
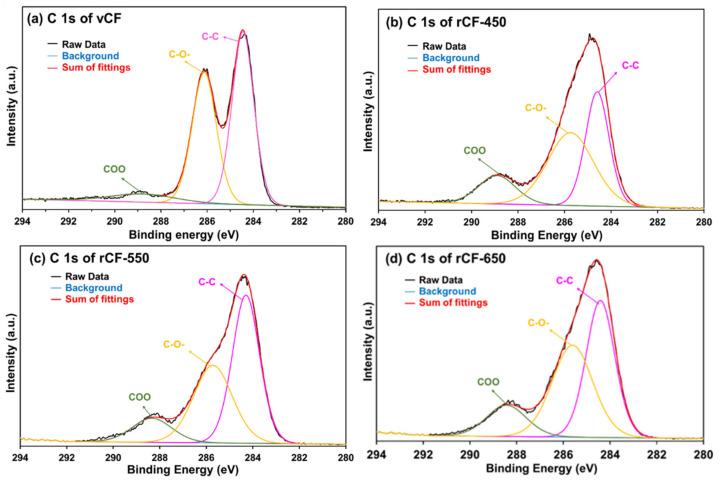
C 1s high resolution spectrum of: (**a**) vCF; (**b**) rCF-450; (**c**) rCF-550; and (**d**) rCF-650.

The functional groups on the fibre surface were determined by C 1s high-resolution narrow spectrum. The C 1s narrow spectra was curve-fitted to show three peaks, namely C–C (284.4 eV) [44], C–O– (285.4–286.3) [44] and COO– (287.2–289.3) [44], according to their distinguished binding energy. The results of C 1 spectra fittings of carbon fibres are shown in Figure 7 and the content of the functional groups are listed in Table 5. The virgin CF has the highest proportion of C–O– group due to the epoxy sizing on the fibre surface, showing consistency with the result of O/C ratio. The recycled fibres have similar functional groups as virgin fibre, but the relative content varied when different temperatures were applied for pyrolysis. Similarly, the major functional group on the surfaces of recovered fibre is the C–C bond, while the oxygen-containing groups (C–O– and COO–) of recovered fibres had higher contents than that of virgin fibre, indicating the chemical reaction between carbon fibre and oxygen took place during the resin decomposition and char oxidation process. rCF-550 had a similar relative content of C–C bond as the virgin fibre. The reduced relative contents of C–C bonds on the surface of rCF-450 and rCF-650 was possibly due to incomplete char removal and over oxidisation, respectively. rCF-650 had the most abundant oxygen-containing group, which was a possible reason for the reduced mechanical properties of rCF-650.

The higher content of oxygen-containing groups on the surfaces of rCFs has been documented in numerous publications. Song et al. attributed the increased oxygen-containing groups to the chemical reaction between carbon fibres and oxygen [39]. Wu et al. suggested that the increased contents of C=O and C–OH on the surface of recycled CF was due to the mild oxidation in the air [45]. Yang et al. also demonstrated that C–C reacted with oxygen to form the oxygen-containing groups by increasing the temperature and oxygen concentration in the mixed gas atmosphere [46]. The increased proportion of oxygen-containing groups would potentially increase the surface activities of rCFs, and consequently enhance the interfacial bonding in a composite reusing the recovered fibres.

### 3.6. Composition of Gaseous Pyrolytic Products

During pyrolysis, gas is produced as a consequence of de-volatilisation, cracking reaction at high temperatures and the secondary reactions between the species released [47,48]. In this study, the gas produced from microwave pyrolysis of CFRP prepregs at different temperature were analysed using GC. As shown in Table 6, the major components include CO (16.64–43.91 vol.%), H_2_ (11.66–47.04 vol.%), CO_2_ (26.84–42.03 vol.%) and CH_4_ (0.51–822 vol.%), while C_2_H_6_, C_2_H_4_ and C_3_H_8_ at much lower contents were also produced. As the pyrolysis temperature raised from 450 to 650 °C, the concentration of H_2_ increased significantly from 11.66 vol.% to 47.04 vol.%, whereas the contents of CO_2_, H_2_ and CH_4_ fluctuated.

The species of gaseous pyrolytic products are comparable with previous studies investigating the pyrolysis of polymer resin. Yang et al. [46] reported that major gas component of pyrolysis of CF/EP composite was CO, CH_4_, H_2_ and CO_2_. Williams et al. found that the major gaseous products of PET pyrolysis were CO_2_ (up to 22.71%) and CO (up to 13.29%) [49]. Torres et al. [50] pyrolyzed SMC composite with a thermoset resin and reported that the gas product was rich in CO (56–68 vol.% and CO_2_ (22–34 vol.%).

**Figure 8:**
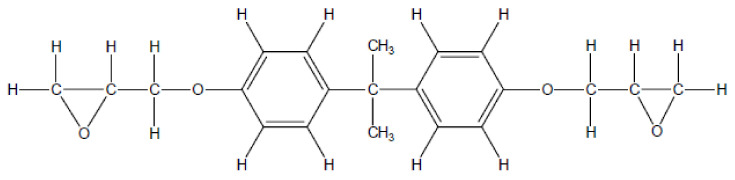
Chemical structure of bisphenol-A epoxy resin [51].

Figure 8 shows the molecular structure of the bisphenol-A epoxy resin typically used in industry [51]. The molecule contains ether bonds (C–O–C) and epoxide groups. Referring to the composition of pyrolytic gas product, CO and CO_2_ are possibly produced from cracking of oxygen heterocycle and ether bonds (C–O–C) within the epoxy molecules, while the hydrocarbon is produced by the breakdown of the alkyl group in the polymer chain of epoxy [52]. H_2_ is produced by the dehydrogenation reactions of char and oil, such as aromatisation, condensation and alkene formation [53]. Moreover, a higher pyrolysis temperature favours the cracking of the volatiles. Cunliffe et al. [54] reported that the CO in the gaseous product of pyrolysis of pure epoxy increased as the pyrolysis temperature raised from 400 to 500 °C. 

### 3.7. Composition of Liquid Pyrolytic Products

The liquid pyrolytic produced under different pyrolysis temperature were characterised using GC/MS. The relative percentage of each class of chemicals and their major compounds were investigated, as listed in Table 7. In general, the components are classified as phenols, aromatics, hydrocarbons, amines, acids and other components. The main products include phenols, aromatics and hydrocarbons. The production of these chemicals was likely due to the breakdown of C–C and C-O bonds presented in the epoxy resin (see Figure 8) being pyrolyzed. As the temperature raised to 650 °C, the phenols increased by around 20 wt.%, indicating that the phenols or benzene derivatives were cracked at high pyrolysis temperatures while the aromatics, hydrocarbons and other components reduced in proportion. Amines were identified in the oil produced and the composition of these reduced with the higher temperatures which are due to the bisphenol A and amine compounds with two aromatic rings that are relatively difficult to remove in oil products [46]. Epoxy resins are essentially aromatic chemicals [55]. Cunliffe et al. reported that the oils produced after the pyrolysis of glass fibre/polyester composites contained aromatic compounds [13]. Torres et al. reported that the oil pyrolytic product of SMC composites contained 64–68% aromatic compounds [50]. In this study, however, the major component of the oil product of the pyrolysis comprised phenols. Similar composition of liquid pyrolytic products (phenols, aromatics and their derivates) from CF/EP composite were previously reported by Yang et al. [46]. Pyrolytic oils are potentially useful as fuel and are possible to be used in the syngas (CO + H_2_) and as such have an identifiable commercial value [55].

## 4. Conclusions

In this work, carbon fibres were extracted from cured CF/EP prepreg successfully under microwave pyrolysis process at the temperature of 450, 550 and 650 °C. Increasing temperature has a positive effect on reducing the char residue on fibre surface as well as increasing the yield of oil and gas products. The carbon fibre recovered at 450 °C showed the highest tensile strength compared to that of other groups due to the char protective effect. The fibre strengths compared favourably with several previous studies. There was no significant change in tensile modulus for all extracted fibre. Some pyrolytic char residue was observed on the recovered fibre surface after microwave pyrolysis. After the oxidation process, the recycled carbon fibre showed a relatively clean and complete surface. XPS indicated that the O/C element ratio increased for the recycled carbon fibre, and the oxygen-containing group increased after the recycling process. For the resin decomposition product, the main components were H_2_, CO and CO_2_ in gaseous phase product and the major liquid components were phenols and aromatics.

With the boosting development of the composite industry, the waste after manufacturing and the end-on-life composite products have become an issue that requires imminent remedial actions to be taken. Recovering the carbon fibres from wastes is gaining more interest, and a cleaner, more productive and more energy-efficient method would always be the pursuit of both researchers and industrialist. The microwave heating investigated in this work showed the potential advantage of selectively heating to the carbon fibres, therefore became much more time-efficient than the conventional techniques that heat the bulk material. Such a heating process is also considered to save energy comparing to other thermal recycling processes.

The scale-up of the current process will be the next challenge. The microwave pyrolysis requires a closed space, and the pyrolyzed materials are then delivered for the subsequent oxidation process. The manner of material feed-in and delivery of materials after each process would be greatly different from the industrialised thermal recycling process. In addition, for the downstream recycled gas and oil, the waste management and disposal system should be well constructed. The energy recovery system from the pyrolyzed oil and gas should also be considered to further reduce energy consumption. In short, the improvement and scale-up of a microwave pyrolysis-oxidation system are attractive, calling for the cooperation of expertise in microwave engineering, dynamic control engineering and energy engineering.

## Figures and Tables

**Table 1:** Experiment schema and test sample code.

*Experiment*	Microwave Pyrolysis			Thermal Oxidation	
	Temperature (°C)	Time (min)		Temperature (°C)	Time (min)
E1	450	15		550	30
E2	550	15		550	30
E3	650	15		550	30
*Sample code*	*Description*				
vCF	Virgin Carbon Fibre				
rCF-450	Recycled Carbon Fibre collected from E1				
rCF-550	Recycled Carbon Fibre collected from E2				
rCF-650	Recycled Carbon Fibre collected from E3				

**Table 2:** Mass balance of microwave pyrolysis of carbon fibre epoxy prepreg.

Temperature (°C)	Solid Residue (wt.%)	Char * (wt.%)	Oil (wt.%)	Gas (wt.%)
450	69.12	6.12	17.71	13.16
550	67.55	4.55	19.15	13.30
650	65.93	2.93	20.28	13.79

* Char is calculated by solid residue minus fibre weight fraction (63 wt.%).

**Table 3:** The tensile properties of virgin and recycled carbon fibre.

Sample	Tensile Strength (MPa)	Tensile Modulus (GPa)	Strength Retention (%)
**vCF**	4670	212.22	100%
**rCF-450**	4078	201.23	87.3%
**rCF-550**	3870	203.57	82.9%
**rCF-650**	3766	196.96	80.6%

**Table 4:** The elemental concentration on the carbon fibre surface determined by XPS.

Samples	Element Composition (at.%)			
	C 1s	O 1s	N 1s	O 1s/C 1s
vCF	77.10	22.39	0.51	0.290
rCF - 450	78.05	19.77	0.97	0.253
rCF - 550	75.53	23.02	1.45	0.305
rCF - 650	74.12	24.57	1.31	0.331

**Table 5:** The relative contents of functional groups on carbon fibre surface determined by XPS.

Sample	Functional Group (%)		
	C–C 284.6 eV [44]	C–O–, C–OH 285.4–286.3 eV [44]	C=O, COO– 287.2–289.3 eV [44]
vCF	50.46	42.52	7.02
rCF-450	45.61	41.37	13.02
rCF-550	50.37	37.67	11.96
rCF-650	44.01	42.04	13.95

**Table 6:** The gas product from microwave pyrolysis of CF/EP prepreg.

Gas (vol.%)	Pyrolysis Temperature (°C)		
	450	550	650
CO	39.68	42.03	26.84
H_2_	11.66	32.18	47.04
CO_2_	43.91	16.64	25.56
CH_4_	4.55	8.22	0.51
C_2_H_6_	0.12	0.57	0.03
C_2_H_4_	0.06	0.25	0.02
C_3_H_8_	0.02	0.12	0.00

**Table 7:** The liquid product composition (wt.%) from microwave pyrolysis of CF/EP prepreg.

Classification	Major Compounds	Temperature (°C)		
		450	550	650
Phenols	Phenol Phenol, 2-methy lPhenol, 3-ethyl p-Cumenol p-Croesl etc.	51.31	53.10	71.69
Aromatics	Benzene Benzene, 1-ethyl-4-methoxy Benzene, 1,3-bis (1,1-dimethylethyl) etc.	16.34	11.57	4.90
Hydrocarbons	Hexane, 2,3,4-trimethyl Dodecane, 4,9-dipropyl Heptadecane, 3-methyl Eicosane etc.	12.19	3.82	5.93
Amines	Benzenamine, 3,4-dichloro Benzenediamine, 4-methyl etc.	5.03	9.89	0.97
Acid	Benzoic acid, 2-(4methylphenoxy) Diphenolic acid etc.	3.04	7.01	0.00
Others	Some Ethanols, Ethers etc.	11.29	14.62	16.53

## Data Availability

The data presented in this study are available on request from the corresponding author.
